# A comprehensive analysis of the role of stem cell transplantation in mantle cell lymphoma: real-world data from the Korean Society of Blood and Marrow Transplantation registry: Stem cell transplantation outcomes in mantle cell lymphoma

**DOI:** 10.1007/s44313-025-00092-4

**Published:** 2025-08-13

**Authors:** Dong Won Baek, Joon Ho Moon, Jae Hoon Lee, Ka-Won Kang, Ho Sup Lee, Hyeon-Seok Eom, Eunyoung Lee, Ji Hyun Lee, Jeong-Ok Lee, Seong Kyu Park, Seok Jin Kim, Youngil Koh, Jong-Ho Won, Jung-Hee Lee, Joon Seong Park, Jae-Cheol Jo, Yeung-Chul Mun, Deok-Hwan Yang, Ga-Young Song, Sung-Nam Lim, Sang Kyun Sohn, the Korean Society of Blood and Marrow Transplantation

**Affiliations:** 1 Department of Hematology/Oncology, Kyungpook National University Hospital, School of Medicine, Kyungpook National University, Daegu, Republic of Korea; 2 Hematology, Gachon University Gil Medical Center, Incheon, Republic of Korea; 3 Division of Hematology-Oncology, Department of Internal Medicine, Korea University College of Medicine, Seoul, Republic of Korea; 4 Division of Hematology, Department of Internal Medicine, Kosin University College of Medicine, Kosin University Gospel Hospital, Busan, Republic of Korea; 5 Department of Hematology-Oncology, Center for Hematologic Malignancy, National Cancer Center, Goyang, Republic of Korea; 6 Division of Hematology-Oncology, Department of Internal Medicine, Dong-A University College of Medicine, Busan, Republic of Korea; 7 Department of Internal Medicine, Seoul National University Bundang Hospital, Seoul National University College of Medicine, Seongnam-si, Republic of Korea; 8 Division of Hematology-Oncology, Department of Internal Medicine, Soonchunhyang University Bucheon Hospital, Bucheon, Republic of Korea; 9 Division of Hematology-Oncology, Department of Medicine, Samsung Medical Center, Sungkyunkwan University School of Medicine, Seoul, Republic of Korea; 10 Department of Internal Medicine, Seoul National University Hospital, Seoul, Republic of Korea; 11 Division of Hematology-Oncology, Department of Internal Medicine, Soonchunhyang University Seoul Hospital, Seoul, Republic of Korea; 12 Department of Hematology, Asan Medical Center, University of Ulsan College of Medicine, Seoul, Republic of Korea; 13 Department of Hematology-Oncology, Ajou University School of Medicine, Suwon-si, Republic of Korea; 14 Department of Hematology and Oncology, Ulsan University Hospital, University of Ulsan College of Medicine, Ulsan, Republic of Korea; 15 Department of Internal Medicine, Ewha Womans University School of Medicine, Seoul, Republic of Korea; 16 Department of Hematology-Oncology, Chonnam National University Hwasun Hospital, Chonnam National University Medical School, Jeollanam-do, Republic of Korea; 17 Division of Hematology-Oncology, Department of Internal Medicine, Inje University College of Medicine, Haeundae Paik Hospital, Busan, Republic of Korea

**Keywords:** Mantle cell lymphoma, Autologous, Allogeneic, Stem cell transplantation, Survival

## Abstract

**Purpose:**

Stem cell transplantation (SCT) has historically played a major role in the long-term remission of mantle cell lymphoma (MCL), an incurable hematological malignancy. Using data from the Korean Society of Bone and Marrow Transplantation registry, we retrospectively analyzed the role of autologous (auto) and allogeneic (allo) SCT in long-term MCL survival.

**Methods:**

This study analyzed data from 188 patients (age ≥ 19 years at the time of transplantation) who underwent a transplant for MCL from 2011 to 2020. Progression-free survival (PFS) was defined as the time from transplantation to disease progression, relapse, or death from any cause. Overall survival (OS) was defined as the time from transplantation to death from any cause or the last follow-up.

**Results:**

In total, 109 patients underwent consolidative SCT after first-line chemotherapy. The 3-year PFS and OS rates were 65.4% and 78.5%, respectively, in the auto-SCT group, and 66.7% and 71.4%, respectively, in the allo-SCT group. The PFS and OS did not differ significantly between the auto- and allo-SCT groups. As part of salvage treatment, 52 patients with relapsed or refractory disease underwent auto- or allo-SCT. Patients who underwent auto-SCT with complete remission/partial remission status reported better outcomes. In patients with refractory status, allogeneic transplantation using human leukocyte antigen (HLA) fully matched donors was a significantly favorable factor for PFS and OS.

**Conclusion:**

The long-term survival of patients who underwent consolidative transplantation was similar to that reported in previous studies. Auto-SCT may be beneficial in patients who respond to salvage therapy, whereas allo-SCT with HLA-matched donors may be an alternative for patients with refractory disease.

**Supplementary Information:**

The online version contains supplementary material available at 10.1007/s44313-025-00092-4.

## Introduction

Mantle cell lymphoma (MCL), which originates from the lymph node mantle zone, accounts for 3%–7% of non-Hodgkin lymphomas [1, 2]. MCL, which may involve the lymph nodes and extranodal sites including the gastrointestinal tract, bone marrow, and blood, presents with various clinical courses. A subgroup of patients with high tumor burden and/or risk factors, such as blastoid morphology, high Ki-67 index, and *TP53* alterations, may require immediate intensive treatment because of the rapidly growing aggressive nature of the disease. However, because such patients have historically shown a poor response to initial therapy, MCL is regarded as incurable [1–3]. 

Generally, rituximab-containing combination regimens, such as rituximab plus cyclophosphamide, doxorubicin, vincristine, and prednisolone (R-CHOP) or bendamustine plus rituximab (BR), are the standard frontline therapies for patients with newly diagnosed MCL requiring systemic treatment [4, 5]. Many studies have shown that patients who receive high-dose chemotherapy followed by autologous stem cell transplantation (auto-SCT) after frontline therapy have better outcomes [4–8]. Salvage treatment remains challenging in patients with relapsed/refractory (R/R) MCL. Bruton’s tyrosine kinase (BTK) inhibitors and anti-CD19 chimeric antigen receptor (CAR) T-cell therapy have demonstrated improved outcomes in patients with R/R and adverse prognostic factors [9–11]. Moreover, allogeneic stem cell transplantation (allo-SCT) may be useful in patients with R/R MCL [12, 13]. Some patients with adverse risk factors, such as *TP53* alterations, can benefit from allo-SCT [14]. Additionally, allo-SCT can be an alternative modality for patients with failed CAR T-cell therapy or BTK inhibitor-based treatment [9, 15].

Recently, the initial treatment strategy for MCL has significantly diversified based on risk stratification at diagnosis, including *TP53* alterations. The introduction of BTK inhibitors as part of the initial treatment and CAR T-cell therapies in R/R settings significantly affects treatment outcomes. However, these advanced therapies are not widely accessible in South Korea. Proper stem cell collection is essential for autologous transplantation. The presence of a suitable donor and overcoming transplant-related mortality are obstacles for allogeneic transplantation. However, auto- or allo-SCT may be a good alternative after chemotherapy, depending on the clinical situation. Here, we retrospectively analyzed the long-term survival outcomes of patients with MCL who underwent auto- or allo-SCT as consolidation or salvage therapy to identify the role of transplantation in the era of various novel agents and cellular therapy.

## Methods

### Patients

Using data from the Korean Society of Blood and Marrow Transplantation (KSBMT) registry, we retrospectively analyzed the data of 188 patients who underwent transplantation for MCL from 2011 to 2020. Patients aged ≥ 19 years at the time of transplantation were included in the study. Most patients received rituximab-containing combination chemotherapy as frontline treatment, and there was no specific institutional policy for determining transplantation. The choice of chemotherapy and conditioning regimen prior to transplantation were made by local physicians at each center. Response assessment was performed according to the Lugano classification using positron emission tomography/computed tomography [16]. Patients’ clinical data were obtained from the KSBMT registry. Clinical data at the time of diagnosis, such as cytogenetic risk factors or the Mantle Cell Lymphoma International Prognostic Index, were insufficient in the registry; thus, it was not available for subgroup analysis. This study was approved by the Institutional Review Board of Kyungpook National University Chilgok Hospital (KNUCH 2025–02-005) and adhered to the Declaration of Helsinki guidelines.

### Statistical analyses

Categorical variables are reported as counts with proportions. Continuous variables are presented as medians with ranges. Fisher’s exact and Wilcoxon rank-sum tests were used for between-group comparisons of categorical and continuous variables, respectively. Progression-free survival (PFS) was defined as the time from transplantation to disease progression, relapse, or death from any cause. Overall survival (OS) was defined as the time between transplantation and death from any cause or last follow-up. PFS and OS probabilities were calculated using the Kaplan–Meier method and compared using the log-rank test. The Cox regression model was used to identify the long-term survival factors. Factors with a p-value of ≤ 0.1 based on univariate analysis were included in the multivariate analysis. Hazard ratios (HRs) and 95% confidence intervals (CIs) were estimated for each factor. Statistical significance was set at *P* < 0.05. Statistical analyses were conducted using R version 4.3.1 (R Foundation for Statistical Computing, Vienna, Austria; http://www.r-project.org).

## Results

### Outcomes of consolidative upfront stem cell transplantation

The patients’ baseline characteristics are summarized in Table 1. Of the 109 patients who received consolidative upfront SCT after first-line chemotherapy, 88 and 21 underwent auto- and allo-SCT, respectively. The median age at the time of SCT was 48.3 years (range: 19–69 years) and 35.4 years (range: 19–61 years) in the autologous and allogeneic transplantation groups, respectively. Compared with patients who underwent auto-SCT, those who underwent allo-SCT were significantly younger (*p* < 0.001). The proportion of patients with complete remission (CR) was higher in the auto-SCT group (79.5%) than in the allo-SCT group (57.1%), whereas the proportion of patients with partial remission (PR) was higher in the allo-SCT group. The incidence of infection within 100 days of transplantation was significantly higher in the allo-SCT group (*p* = 0.001).

**Table 1:** Baseline characteristics of the patients who underwent upfront stem cell transplantation

Characteristics	Auto-SCT (*N* = 88)	Allo-SCT (*N* = 21)	*p*-value
Age, median (range), years	48.3 (19–69)	35.4 (19–61)	< 0.001
Sex			0.999
Female, no. (%)	31 (35.2)	7 (33.3)	
Male, no. (%)	57 (64.8)	14 (66.7)	
Status			0.064
CR, no. (%)	70 (79.5)	12 (57.1)	
PR, no. (%)	18 (20.5)	9 (42.9)	
Infection (post-100 days), no. (%)	22 (25.0)	14 (66.7)	0.001
Relapse, no. (%)	19 (21.6)	1 (4.8)	0.140
Death, no. (%)	19 (21.6)	6 (28.6)	0.693
Post 100 days, no. (%)	2 (2.3)	2 (9.5)	0.346
Post 1 year, no. (%)	9 (10.2)	4 (19.0)	0.132

*Allo-SCT* allogeneic stem cell transplantation, *auto-SCT* autologous stem cell transplantation, *CR* complete remission, *PR* partial remission

**Fig. 1:**
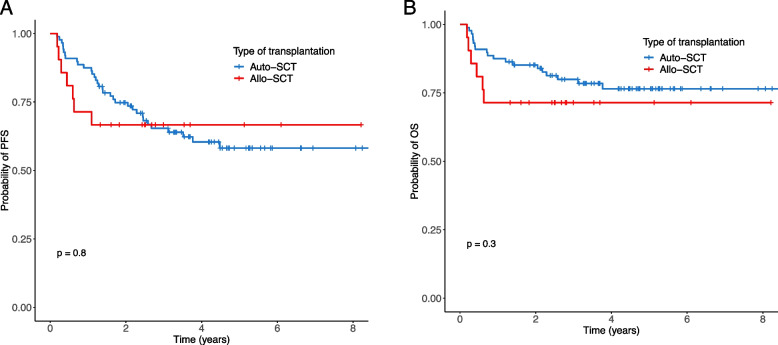
Kaplan–Meier curves. Progression-free survival (**A**) and overall survival (**B**) were not significantly different between the auto- and allo-SCT groups

The median follow-up duration was 5.3 years (range: 0.2–8.8 years). In the auto-SCT group, 19 patients (21.6%) relapsed with a median time of 20.6 months (range: 13.2–54.5 months), and 19 patients (21.6%) died. The 3-year PFS and OS rates were 65.4% and 78.5%, respectively. In the allo-SCT group, one patient (4.8%) relapsed, and six patients (28.6%) died. The 3-year PFS and OS rates were 66.7% and 71.4%, respectively. Kaplan–Meier curves revealed no significant differences in PFS and OS in patients who underwent auto-SCT versus those who underwent allo-SCT (Fig. 1). Although the relapse frequency was higher in the auto-SCT group, early death was higher in the allo-SCT group (Supplementary Fig. 1).

### Outcomes of stem cell transplantation in the salvage setting

**Fig. 2:**
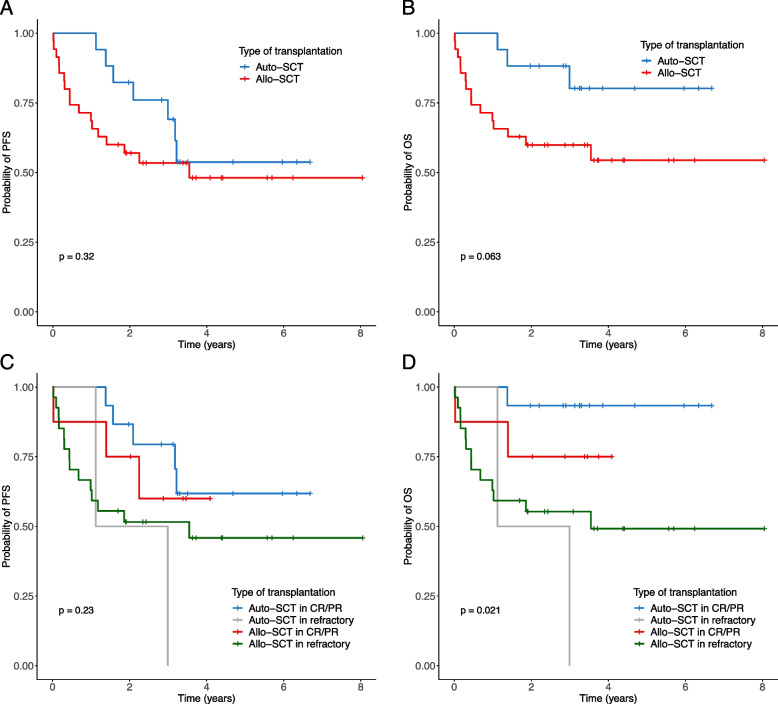
Kaplan–Meier curves. Progression-free survival (PFS) was not significantly different between the auto- and allo-SCT groups (**A**). However, compared with those who underwent allo-SCT, patients who underwent auto-SCT tended to have better overall survival (OS) (**B**). A better PFS trend was confirmed when transplantation was performed with the CR/PR status after salvage therapy (**C**), and patients who received auto-SCT with CR/PR status showed better OS (**D**)

As part of salvage treatment, 52 patients with relapsed or refractory disease underwent auto- (17 patients) or allo-SCT (35 patients). The clinical characteristics of the patients are shown in Table 2. In the autologous and allogeneic transplantation groups, the median age at transplantation was 47.1 years (range: 21–63 years) and 40.6 years (range: 20–61 years), respectively. In the auto-SCT group, eight patients achieved CR, seven exhibited PR at the time of transplantation, and two had a refractory status. In the allo-SCT group, six patients achieved CR, two had PR, and 27 had a refractory status. The incidence of infection within 100 days of transplantation was significantly higher in the allo-SCT group (*p* = 0.014). The median follow-up duration was 2.9 years (range: 0.1–8.1 years). In the auto-SCT group, seven patients (41.2%) relapsed at a median time of 25.4 months (range: 13.6–39.1 months), and three (17.6%) died. The 3-year PFS and OS rates were 68.1% and 78.2%, respectively. In the allo-SCT group, 3 patients (8.6%) relapsed and 15 (42.9%) died. The 3-year PFS and OS rates were 53.4% and 59.9%, respectively. Although the relapse rate was higher in the auto-SCT group, the early mortality rate within 1 year was higher in the allo-SCT group (Supplementary Fig. 2). Overall, the Kaplan–Meier curves revealed no significant differences in PFS between the auto-SCT and allo-SCT groups. However, the patients who underwent auto-SCT had a superior OS rate (Fig. 2A, 2B). In particular, patients who underwent auto-SCT with CR/PR status reported better outcomes than those in the other groups (Fig. 2C, 2D). In the allo-SCT group, 13 patients received prior autologous transplantation, of whom 1 died within 100 days and 3 died within one year after allo-SCT. Three patients experienced relapses. Multivariate analysis revealed that in the salvage setting, the disease status at the time of transplantation was the only factor affecting OS (Supplementary Table 1).

**Table 2:** Baseline characteristics of the patients who underwent stem cell transplantation in the salvage setting

Characteristics	Auto-SCT (*N* = 17)	Allo-SCT (*N* = 35)	*p*-value
Age, median (range), years	47.1 (21–63)	40.6 (20–61)	0.069
Sex			0.624
Female, no. (%)	4 (23.5)	9 (25.7)	
Male, no. (%)	13 (76.5)	26 (74.3)	
Status			< 0.001
CR2, no. (%)	7 (41.2)	6 (17.1)	
CR3, no. (%)	1 (5.9)	0	
PR, no. (%)	7 (41.2)	2 (5.7)	
Refractory, no. (%)	2 (11.8)	27 (77.1)	
Prior auto-SCT, no. (%)	1 (5.9%)	13 (37.1)	0.040
Infection (post-100 days), no. (%)	2 (11.8)	18 (51.4)	0.014
Relapse, no. (%)	7 (41.2)	3 (8.6)	0.015
Death, no. (%)	3 (17.6)	15 (42.9)	0.138
Post 100 days, no. (%)	0	5 (14.3)	0.255
Post 1 year, no. (%)	0	12 (34.3)	0.176

*Allo-SCT *allogeneic stem cell transplantation, *auto-SCT* autologous stem cell transplantation, *CR *complete remission, *PR* partial remission

### Outcomes of patients who received allogeneic transplantation with refractory status

Among patients who underwent allo-SCT in the salvage setting, 27 had refractory disease. The patient characteristics are shown in Table 3. The median age at the time of transplantation was 44.6 years (range: 20–63 years). All patients received peripheral blood stem cells, with 18 patients (66.7%) receiving stem cells from related donors. There were fifteen human leukocyte antigen (HLA) fully matched (55.6%), seven haploidentical (25.9%), and five mismatched (18.5%) donors. Nine patients (33.3%) received a myeloablative conditioning regimen and two (7.4%) received total body irradiation. Infection was confirmed in more than half of the patients within 100 days of transplantation. Ten (37.0%) and nine (33.3%) patients had acute and chronic graft-versus-host disease, respectively. Two patients (7.4%) experienced disease relapse. There were 14 deaths (51.9%), with 10 deaths (37.0%) within a year of transplantation. The 3-year PFS and OS rates were 51.6% and 55.3%, respectively. Multivariate analysis revealed that the use of HLA fully matched donors was a significantly favorable PFS and OS factor (Table 4).

**Table 3:** The characteristics of patients who received allogeneic stem cell transplantation with refractory disease status

Characteristics	N = 27
Age, median (range), years	44.6 (20–63)
Sex	
Female, no. (%)	8 (29.6)
Male, no. (%)	19 (70.4)
Donor type	
Related	18 (66.7)
Unrelated	9 (33.3)
HLA matched	
Full matched	15 (55.6)
Mismatched	5 (18.5)
Haploidentical	7 (25.9)
Intensity of conditioning	
Myeloablative	9 (33.3)
Reduced intensity	18 (66.7)
Total body irradiation	2 (7.4)
Acute GVHD	10 (37.0)
Overall grade of acute GVHD^*^	
I	5 (18.5)
II	2 (7.4)
III	2 (7.4)
IV	1 (3.7)
Chronic GVHD	9 (33.3)
Global severity of chronic GVHD^#^	
Mild	3 (11.1)
Moderate	5 (18.5)
Severe	1 (3.7)
Prior auto-SCT, no. (%)	11 (40.7)
Infection (post-100 days), no. (%)	15 (55.6)
Relapse, no. (%)	2 (7.4)
Death, no. (%)	14 (51.9)
Post 100 days, no. (%)	4 (14.8)
Post 1 year, no. (%)	10 (37.0)

*GVHD* graft-versus-host disease, *HLA* human leukocyte antigen, *SCT* stem cell transplantation

^*^MAGIC criteria

^#^NIH Global Severity

**Table 4:** The factors affecting the long-term survival of refractory patients who underwent allogeneic stem cell transplantation.

	Univariate			Multivariate		
	HR	95% CI	*p*-value	HR	95% CI	*p*-value
1) Progression-free survival						
Age, < 40 vs. ≥ 40 years	3.257	0.473–22.448	0.231			
Sex, male vs. female	0.711	0.172–3.116	0.651			
Prior SCT, none vs. performed	0.988	0.182–5.355	0.989			
Donor, related vs. unrelated	0.223	0.037–1.351	0.102			
HLA, fully matched vs. mismatched	0.054	0.004–0.731	0.028	0.322	0.105–0.993	0.049
Conditioning, MAC vs. RIC	2.151	0.277–16.711	0.464			
Infection within 100 days, yes vs. none	8.264	0.547–124.872	0.127			
Acute GVHD, yes vs. none	6.421	0.704–58.585	0.099	9.871	0.628-64.435	0.107
Chronic GVHD, yes vs. none	0.107	0.012–0.947	0.046	0.302	0.010-1.078	0.065
2) Overall survival						
Age, < 40 vs. ≥ 40 years	4.430	0.451–43.544	0.201			
Sex, male vs. female	0.567	0.126–2.537	0.458			
Prior SCT, none vs. performed	0.331	0.042–2.557	0.289			
Donor, related vs. unrelated	0.377	0.648–2.199	0.279			
HLA, fully matched vs. mismatched	0.020	0.001–0.687	0.030	0.231	0.064–0.828	0.025
Conditioning, MAC vs. RIC	2.527	0.231–27.688	0.448			
Infection within 100 days, yes vs. none	28.063	0.864–910.945	0.060	30.341	0.681-841.547	0.209
Acute GVHD, yes vs. none	14.336	0.801–256.437	0.070	18.756	0.811-314.659	0.227
Chronic GVHD, yes vs. none	0.020	0.001–0.390	0.009	0.291	0.065–1.304	0.107

*CI* confidence interval, *GVHD* graft-versus-host disease, *HLA* human leukocyte antigen, *HR* hazard ratio, *MAC* myeloablative conditioning, *RIC* reduced-intensity conditioning, *SCT* stem cell transplantation

## Discussion

This study reported the long-term survival outcomes of patients with MCL who received transplantation as part of consolidation or salvage therapy using data registered in the KSBMT registry over the past 10 years. Overall, while the relapse frequency was higher in the auto-SCT group, early death was more frequent in the allo-SCT group. There were no significant survival differences between patients who underwent consolidative auto-SCT and those who underwent allo-SCT. In the salvage setting, survival outcomes varied according to the patient’s condition at the time of transplantation. Auto-SCT was associated with superior OS in patients who responded to salvage chemotherapy and achieved CR or PR. It was confirmed that allo-SCT may be effective in chemo-refractory patients and that the use of HLA-matched donors is a favorable prognostic factor.

In young and healthy transplantation-eligible patients, upfront auto-SCT was considered after initial chemotherapy, before BTK inhibitors were actively used as first-line and maintenance therapies. Our data showed that in patients who received upfront auto-SCT, the 3-year PFS and OS rates were 65.4% and 78.5%, respectively, which were similar to the results of previous studies, and there was no survival benefit in patients who underwent allo-SCT. A retrospective review from the National Comprehensive Cancer Network’s non-Hodgkin lymphoma database showed that the 3-year PFS and OS rates of patients who received R-CHOP followed by high-dose therapy with auto-SCT were 56% and 87%, respectively, which were significantly higher than those of patients who received R-CHOP alone [4]. Similar results have been reported for RB followed by auto-SCT (81% and 87% 2-year PFS and OS rates, respectively) [5]. Compared with those who received R-CHOP or RB and underwent auto-SCT, patients who did not undergo transplantation did not exhibit superior survival outcomes despite receiving an aggressive induction regimen (rituximab plus hyperfractionated cyclophosphamide, vincristine, doxorubicin, and dexamethasone, alternating with high-dose cytarabine and methotrexate) [4, 5, 17]. However, the role of upfront consolidative auto-SCT has become ambiguous with the recent introduction of BTK inhibitors as a first-line therapy. In the TRIANGLE trial, patients who underwent autologous transplantation did not exhibit a survival benefit [18]. Although consideration of long-term outcomes and real-world data is needed, the role of upfront transplantation in patients with newly diagnosed MCL is likely limited to settings in which BTK inhibitors cannot be used in combination with immunochemotherapy or for maintenance.

The treatment of patients with R/R MCL remains challenging. In this study, the 3-year PFS and OS rates of patients who underwent auto-SCT with CR/PR status were 68.1% and 78.2%, respectively, but 41.2% of the patients experienced relapse during the follow-up period. Allo-SCT may be an alternative for patients who do not respond to salvage therapy. Only two patients with an R/R status exhibited relapse, suggesting that long-term remission can be achieved if early mortality after transplantation is overcome. BTK inhibitors have recently been reported to improve outcomes, and BTK inhibitor-containing therapy has been established as a standard treatment [9, 10, 19]. However, 17p deletions or *TP53* aberrations are strongly associated with treatment resistance, including to ibrutinib, and patients with blastoid morphology, a high tumor proliferation index, and disease progression within 24 months have exhibited adverse outcomes with ibrutinib monotherapy or ibrutinib-containing combination therapy [20–22]. Additionally, ibrutinib has well-described toxicities and is discontinued by a significant number of patients because of drug intolerance [23]. Brexucabtagene autoleucel and lisocabgene maraleucel have demonstrated promising clinical outcomes [24, 25]. However, the economic burden is high and access to both CAR T-cell therapies is considerably limited. Instead, allo-SCT may be considered in patients with adverse prognostic factors. Previous studies have reported the graft-versus-lymphoma effect of allogeneic transplantation as a means of overcoming the adverse factors of R/R MCL [13, 26]. A study focused on *TP53* alterations and allo-SCT found no significant differences between patients with and without *TP53* alterations when reduced intensity or nonmyeloablative conditioning was used [14].

The results of the current study should be interpreted cautiously as this study used retrospective cohort data with inherent limitations. First, detailed baseline characteristics such as the Mantle Cell Lymphoma International Prognostic Index and cytogenetic risk stratification, which substantially influence prognosis and treatment decisions, were insufficient for analysis. Second, the criteria for selecting auto- or allo-SCT as consolidation therapy were not mentioned. Third, detailed information regarding the frontline therapies and salvage chemotherapies used, including the exposure to and response rates to BTK inhibitors, was not adequately addressed. In addition, detailed conditioning regimen and graft-versus-host disease prophylaxis were not described. Finally, because only patients who underwent a transplant were analyzed, the outcomes of patients with and without transplantation could not be compared. 

In conclusion, our data showed that the long-term survival of patients who underwent consolidative upfront transplantation was consistent with that reported in previous studies and that it might be a good option in certain situations where BTK inhibitors cannot be used as frontline therapy. In R/R settings, autologous transplantation may be beneficial in patients who respond to salvage therapy. For patients with refractory disease, allo-SCT using an HLA fully matched donor may be an alternative, although the risk of transplant-related mortality is a hurdle. Currently, advanced therapies are not widely accessible in South Korea. However, selecting an appropriate stem cell transplantation after chemotherapy may help improve survival outcomes in patients with MCL. 

## Supplementary Information

Supplementary material 1.

## Data Availability

No datasets were generated or analysed during the current study.
