# Supplementary material for: A comprehensive analysis of the role of stem cell transplantation in mantle cell lymphoma: real-world data from the Korean Society of Blood and Marrow Transplantation registry: Stem cell transplantation outcomes in mantle cell lymphoma
Source: Blood Res. 2025 Aug 13;60(1):44. doi: 10.1007/s44313-025-00092-4 (PMC12350992; doi:10.1007/s44313-025-00092-4)
Supplement: Supplementary file 1 — Supplementary material 1. [file 44313_2025_92_MOESM1_ESM.docx]

**Supplementary Fig. 1.** The cumulative relapse (A) and mortality (B) rates of consolidative autologous and allogeneic transplantation.

(A)


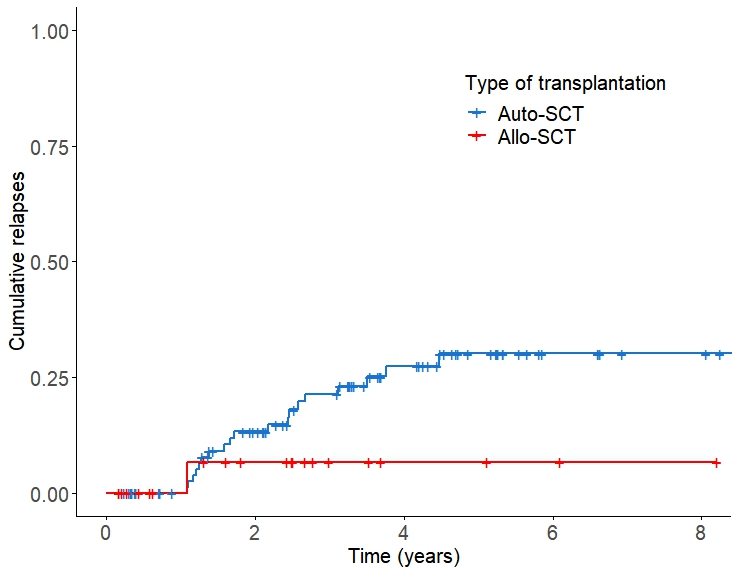


(B)


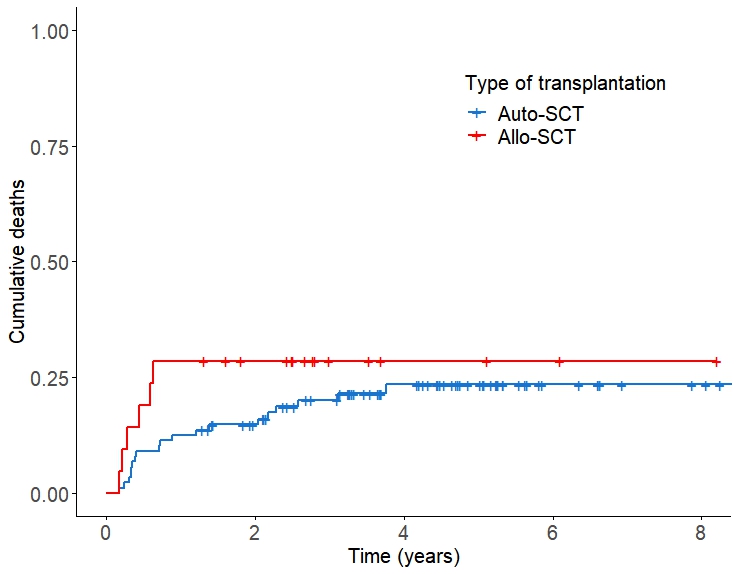


**Supplementary Fig. 2.** The cumulative relapse (A) and mortality (B) rates of autologous and allogeneic transplantation in the salvage setting.

(A)


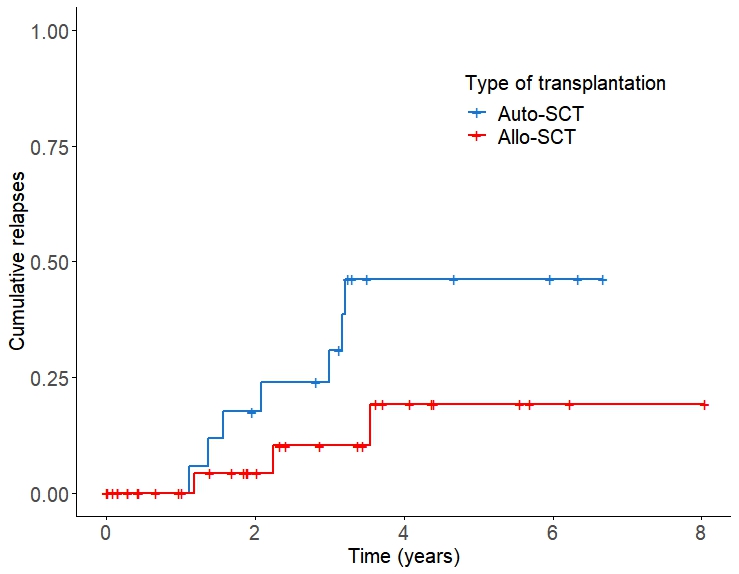


(B)


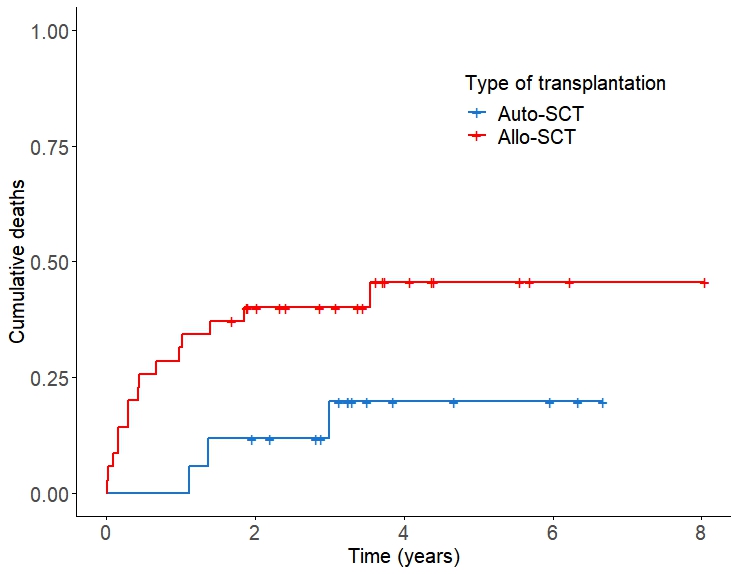


**Supplementary Table 1.** The factors affecting long-term survival outcomes.

1) Progression-free survival

|  | Univariate | | |  | Multivariate | | |
| --- | --- | --- | --- | --- | --- | --- | --- |
|  | HR | 95% CI | p-value |  | HR | 95% CI | p-value |
| Age, < 40 vs. ≥ 40 | 0.794 | 0.523–4.632 | 0.427 |  |  |  |  |
| Sex, male vs. female | 0.969 | 0.205–1.711 | 0.333 |  |  |  |  |
| Status, refractory vs. CR/PR | 1.527 | 0.771–8.097 | 0.127 |  |  |  |  |
| Prior SCT, performed vs. none | 1.292 | 0.727–4.707 | 0.196 |  |  |  |  |
| SCT type, autologous vs. allogeneic | 0.437 | 0.192–2.846 | 0.662 |  |  |  |  |

2) Overall survival

|  | Univariate | | |  | Multivariate | | |
| --- | --- | --- | --- | --- | --- | --- | --- |
|  | HR | 95% CI | p-value |  | HR | 95% CI | p-value |
| Age, < 40 vs. ≥ 40 | 0.086 | 0.325–3.414 | 0.931 |  |  |  |  |
| Sex, male vs. female | 0.680 | 0.201–2.179 | 0.496 |  |  |  |  |
| Status, refractory vs. CR/PR | 1.919 | 0.967–23.392 | 0.055 |  | 2.545 | 1.449–17.391 | 0.011 |
| Prior SCT, performed vs. none | 1.472 | 0.454–3.633 | 0.637 |  |  |  |  |
| SCT type, autologous vs. allogeneic | 0.002 | 0.184–5.445 | 0.998 |  |  |  |  |

CI: confidence interval, CR: complete remission, HR: hazard ratio, PR: partial remission, SCT: stem cell transplantation.
